# Supplementary material for: Comparison of marker-less 2D image-based methods for infant pose estimation
Source: Sci Rep. 2025 Apr 9;15:12148. doi: 10.1038/s41598-025-96206-0 (PMC11982382; doi:10.1038/s41598-025-96206-0)
Supplement: Supplementary file 1 — Supplementary Information. [file 41598_2025_96206_MOESM1_ESM.pdf]

A Appendix

A.1 Keypoint detection rates

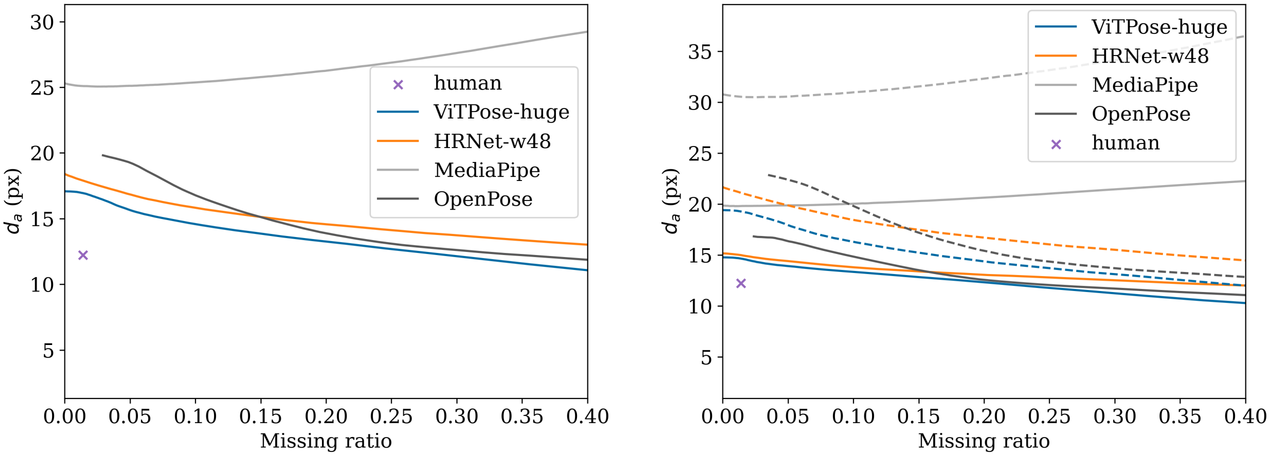

(a) Overall error **(b)** Split by viewing angle

Figure S1. Mean difference to annotation d_a vs. the ratio of missing detections, based on the keypoint prediction confidence scores. Panel a): overall, panel b): split by viewing angle, solid lines correspond to the top view, dashed lines to the diagonal view.

This section contains an additional analysis of the models own estimation of reliability. All models also score the predictions with a certainty value c between 0 and 1, e.g., how confident the model is in its prediction. This value in itself is not normalized between the models. To enable comparison, we do not look at these certainty values, but at the level of missing keypoints. To obtain it, we thresholded the certainty values, for thresholds t between 0 and 1, and then calculated the respective ratio of missing predictions m as

$$m = \frac{1}{N}\sum_{i=1}^{N} H(c-t).$$

There, H is the Heaviside function and N the total number of annotated keypoints in the dataset. This effectively normalizes the score to the percentage of points deemed correct by the model itself. We then plotted d_a against this dependent value m instead of the model output c.

The keypoint detection rates for all models are displayed in Figure S1a. The result for the human annotation variance (see Section 3.1) was also added for comparison.

Except for MediaPipe, the error decreases when filtering out uncertain points. The increase for MediaPipe comes from the model assigning relatively high scores to the ears, which are among its worst detected points. ViTPose-huge has the lowest error across all missing ratios, MediaPipe the highest. OpenPose achieves a lower error than HRNet for missing ratios over 15%, but this is of limited use, as ViTPose is still better and 15% of points not being detected is insufficient for the applications of automated GMA. Moreover, OpenPose does not even detect all points when thresholding with a score of 0, resulting in the missing ratio never dropping below 2.93%. ViTPose and HRNet, however, produce predictions for every possible point when thresholding at 0. Below the missing ratio for humans (1.41%, because of the ears), the error of ViTPose saturates, while the error of HRNet increases. This is because ViTPose is assigning low scores to the ear keypoints that are not visible (e.g., due to head turned to the side) and therefore don't affect the error calculation.

Figure S1b shows the mean difference to annotation in dependence of the missing ratio, like Figure S1a, but split by perspective. The performance for the diagonal view is always worse than for the top view. Moreover, while the error for the diagonal view drops faster than for the top view when filtering out uncertain points, they don't converge to the same level even when filtering out more than 20% of all detected points. We again observe OpenPose not being able to provide estimates for every point, with the diagonal angle having more non-detected keypoints as compared to the top view.

In summary, filtering out points with low certainty scores yields better results in terms of pose estimation error (d_a) for all models but MediaPipe. However, this is of limited value for practical use, since, for most applications, missing values would have to be filled in by interpolation, median filtering, or other techniques (e.g., Kalman filter), to be used for motion analysis.

Still, the fact that the least certain points for ViTPose are the ones humans could not annotate, manifesting in stagnating d_a for low missing ratios, shows the certainty score aligns with actual visibility constraints for the state-of-the-art model.

A.2 SyRIP

The SyRIP dataset[40] was considered, and some preliminary analyses were performed, however, it became obvious that the setting of this dataset is too different from GMA setting, with many more different body positions and much older infants than in our dataset. As in case of the specialized infant pose estimators evaluated on our dataset, our model could not compete with the generic ViTPose on SyRIP. Retraining decreased the PCK@0.05 from 51.53% to 27.65% (73.66% to 46.97% for PCK@0.1), further strengthening our point that specialized infant pose estimators (in this case our own) are overfit and do not generalize well to other datasets.
